# Evaluating the Effectiveness of the COVID-19 Emergency Outbreak Prevention and Control Based on CIA-ISM

**DOI:** 10.3390/ijerph19127146

**Published:** 2022-06-10

**Authors:** Renlong Wang, Endong Wang, Lingzhi Li, Wei Li

**Affiliations:** 1 Research Center of Smart City, Nanjing Tech University, Nanjing 211816, China; 13127073530@163.com; 2 College of Civil Engineering, Nanjing Tech University, Nanjing 211816, China; 3 Department of Sustainable Resources Management, State University of New York, Syracuse, NY 13210, USA; 4 Department of Construction and Real Estate, School of Civil Engineering, Southeast University, Nanjing 211189, China; 230179099@seu.edu.cn

**Keywords:** CIA-ISM, COVID-19, emergency management, scenario analysis, scenario deduction

## Abstract

The COVID-19 pandemic, characterized by high uncertainty and difficulty in prevention and control, has caused significant disasters in human society. In this situation, emergency management of pandemic prevention and control is essential to reduce the pandemic’s devastation and rapidly restore economic and social stability. Few studies have focused on a scenario analysis of the entire emergency response process. To fill this research gap, this paper applies a cross impact analysis (CIA) and interpretive structural modeling (ISM) approach to analyze emergency scenarios and evaluate the effectiveness of emergency management during the COVID-19 crisis for outbreak prevention and control. First, the model extracts the critical events for COVID-19 epidemic prevention and control, including source, process, and resultant events. Subsequently, we generated different emergency management scenarios according to different impact levels and conducted scenario deduction and analysis. A CIA-ISM based scenario modeling approach is applied to COVID-19 emergency management in Nanjing city, China, and the results of the scenario projection are compared with actual situations to prove the validity of the approach. The results show that CIA-ISM based scenario modeling can realize critical event identification, scenario generation, and evolutionary scenario deduction in epidemic prevention and control. This method effectively handles the complexity and uncertainty of epidemic prevention and control and provides insights that can be utilized by emergency managers to achieve effective epidemic prevention and control.

## 1. Introduction

As a public health emergency, the outbreak of the COVID-19 pandemic has resulted in massive impacts on human society globally [1]. First, the COVID-19 pandemic spread over a wide range of regions and seriously endangered public health and human lives. The highly contagious nature of COVID-19 virus caused an instantaneous spike in the scale of population who became ill in a short period. It was estimated that without policy interventions, the infection rate of COVID-19 could have grown exponentially at 38% each day in some countries, such as China and the US [2]. A high level of tension in health care workers and dramatic shortages in the medical supply chain were extensively reported. Due to the supply chain issue, personal protective equipment and necessary testing kits for medical treatment and diagnosis became a significant challenge for many countries [3]. Serious psychological panic occurred, affecting the regular operation of economic organizations and social order. Moreover, the outbreak of the COVID-19 pandemic triggered a “domino” effect, as the pandemic affected many other pillar industries, such as construction, tourism, aviation, and so forth. A direct consequence of this chain effect was the complication of social disorder crisis. These disruptive effects forced the prevention and control of COVID-19 crisis to become a common concern worldwide.

Scenario analysis has been frequently used for emergency management and crisis analysis by academic researchers. Based on both existing certain and potential uncertain event conditions, scenario modeling can create future possible situations for pathway analysis to identify suitable measures for controlling negative outcomes [4]. Application examples of scenario analysis for emergency management include scenario modeling for emergency preparedness [5], effectiveness of earthquake emergency management [6], logistics preparation during flood emergency [7], and hurricane disaster emergency responses [8]. Similar to these previous emergency scenarios, many complex factors or events, including uncertain objective factors beyond human control (e.g., mutation of COVID-19, timing and location of the outbreak) and subjective factors due to serious negligence in risk prevention (e.g., untimely release of epidemic information, inadequate public education) can affect the outbreak and spread of the COVID-19 crisis [9]. These factors or events are generally dynamic, and their occurrences are often urgent and interdependent on each other. In addition, the region where a COVID-19 outbreak occurs presents a high degree of dynamic openness, making COVID-19 more complex and more uncertain than other emergency events, and the decisions for pandemic prevention and control are more difficult. In this context, scenario modeling can be a critical tool to provide a structured approach for COVID-19 emergency management and control. Therefore, scenario analysis of the effectiveness of COVID-19 prevention and control can provide managers with a multidimensional and comprehensive understanding of epidemic emergency management, including critical scenario identification, scenario logic relationships, scenario evolution, and so on.

The combination of cross impact analysis and interpretive structural modeling (CIA-ISM) is a comprehensive scenario analysis method that can generate future scenarios and analyze their developments based on occurrence possibilities of identified events and their interdependencies [4]. It can clearly establish causal relationships between factors or events in dynamic situations by considering their cross-impacts for the identified time horizon. Recently, CIA-ISM has been utilized by several studies for the purpose of managing emergencies and risks [5,10], such as industrial operation risks, earthquake emergency, etc. [6,10]. CIA is used for analyzing the interaction impacts between events or factors of an emergency process by applying an analogy between causality and atomic excited states [11]. ISM can present possible emergency scenarios and the interdependencies between events based on cross-impacts and reachability. This tool can assist managers and stakeholders to more clearly envision future scenarios, as well as their development, and make decisions for emergency management. According to [5], CIA-ISM can be used for the management of any emergency process for planning support. It can identify and display critical events based on cross-impacts and structured graphing systems. Both direct and indirect relationships among events can be presented by CIA-ISM. Notably, the CIA-ISM method is based on the Delphi method and is more suitable for causal logic analysis and scenario inference between macro events for which no objective quantitative data are available, and therefore, the development of the CIA-ISM model in this paper does not involve the relevant attributes of specific COVID-19 viruses.

Many scholars have researched pandemic prevention and control management in response to the COVID-19 emergency. For example, some scholars have suggested preventive measures for medical professionals from the perspective of the originating stages of the COVID-19 outbreak [12]. Some scholars analyzed the development of the COVID-19 outbreak and made recommendations for ensuring human safety and psychological well-being, and to facilitate regional stability [13,14,15]. Other scholars have analyzed the causes of the COVID-19 outbreak and summarized successful experiences to combat the pandemic [16,17]. The above studies have mainly focused on a specific aspect of COVID-19 outbreak prevention and control management but failed to effectively address the dynamic and interdependent nature of critical events in COVID-19 emergency management. In addition, these studies failed to quantify the importance and logical relationships of the critical events of COVID-19 outbreak prevention and control from a global perspective (i.e., the origin, development and outcome of the whole pandemic management process), which is of limited help to non-specialist government decision makers. To fill these gaps, this paper applies a CIA-ISM model to develop a COVID-19 prevention and control model to quantitatively assess the effectiveness of outbreak emergency management and provide managers with a deep understanding of the critical points of COVID-19 prevention and control from a dynamic and interrelated logical perspective. 

Our study aims to: (1) identify the important events that can affect COVID-19 outbreak and spread, (2) estimate the interdependency between these COVID-19 related events, (3) apply a scenario analysis model to simulate the evolution of a COVID-19 epidemic by cross-impacts, and (4) provide government agencies with suitable strategies to improve the performance of epidemic prevention and control in a COVID-19 crisis. To achieve the above objectives, this paper applies a CIA-ISM scenario-based approach to evaluate the effectiveness of emergency prevention and control actions during the COVID-19 outbreak and spread. First, the critical events of epidemic prevention and control in emergency management are selected to form the initial event set. Then, we invited experts from public health management fields to estimate the probability of the identified events and their interactions. These estimation data are entered into the CIA-ISM model for scenario generation, critical event identification, and pathway deduction. Finally, for feasibility testing, the CIA-ISM model is applied to COVID-19 emergency management in Nanjing city, China. 

The contributions of this paper are summarized as the following:

(1) It extends the application of CIA-ISM to COVID-19 emergency for epidemic prevention and control.
(2) It realizes the integration of expert knowledge from multiple relevant fields into COVID-19 emergency management.
(3) It realizes emergency control through a multi-dimensional analysis of the origin, development, and outcome of the whole epidemic management process and the structured and causal presentation of epidemic emergency management scenario.

The remaining parts of this paper are organized as follows: Section 2 introduces the research process of the CIA-ISM approach. Section 3 presents the application of CIA-ISM to COVID-19 emergency management. In Section 4, a case study of COVID-19 emergency management in Nanjing, China is constructed to demonstrate the feasibility of the proposed approach. Section 5 discusses applications of the CIA-ISM method and its intrinsic logic and makes recommendations for emergency management of COVID-19 outbreak prevention and control. Finally, Section 6 provides conclusions and future research directions.

## 2. Research Method

**Figure 1:**
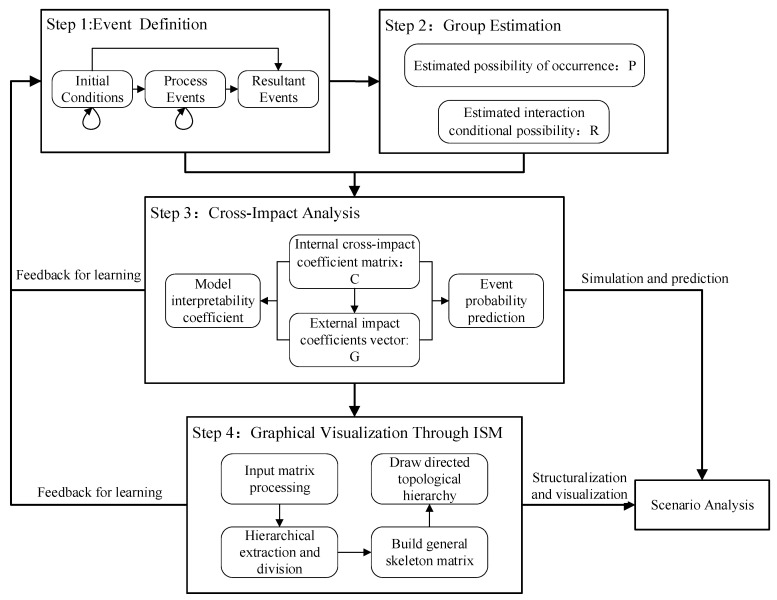
The process of CIA-ISM.

The CIA-ISM method for scenario construction, impact analysis, and consequence prediction is presented in this section. As shown in Figure 1, the process of CIA-ISM includes four steps: event identification, group estimation, cross-impact analysis, and graphical visualization through ISM.

### 2.1. Step 1: Event Definition

An event represents the basic element unit on which the CIA-ISM method operates to construct scenarios, analyze interdependencies, and forecast future dynamics. By breaking down the targeted emergency course into small event and process units, a set of events that are critical to the emergency problem under consideration can be identified. Based on Bañuls and Turoff (2011) and Bañuls et al. (2013), the extracted events are classified into three categories: source events, process events, and resultant events [4,5]. This event identification process can be completed by relevant literature analysis or a group-based brainstorming meeting to collect the ideas or viewpoints of relevant experts. To establish a high-quality event set that can accurately represent the studied emergency process, the experts who have knowledge about the emergency events are interviewed for event identification.

Source Events ($SE_{i}$): Source events represent important initial conditions and scenario assumptions that have occurred (or not) before the studied time period when an emergency process occurs [4,5]. They are selected to describe the existing conditions for the emergency. They are supposed to have significant potential impacts on subsequent process and resultant events. The occurrences of source events are only influenced by possible interactions between source events. The initial occurrence possibilities of source events are determined to be 0.5, meaning that occurrence and non-occurrence of the source event has the same probability. Notably, setting the initial probability of the source event to 0.5 is more similar to the initialization of the model, which will be reassigned based on the collected data in subsequent practical applications.

Process Events ($PE_{i}$): Process events represent a chain of core events that occur during an emergency process [4,5]. They are selected to reflect the critical behavior characteristics of different emergency stakeholders in responding to the emergency process. The stakeholders’ responses can be passive perturbations caused by the emergency or active reactions to the emergency, such as how governmental officials handled the situation or public opinions. As with the source events, the estimates for the possibility of these process events are initially determined to be 0.5.

Resultant Events ($RE_{i}$): Resultant events represent final results caused by the emergency, reflecting the combined effects and consequences of source events and process events [4,5]. Therefore, resultant events do not have any impacts on other events. Similar to process events, to obtain estimates for the certainty of resultant events in scenario modeling, the initial probability of resultant event occurrence is determined to be 0.5.

Besides the three types of events mentioned above, the external environment is also defined. The external environment refers to the conditions that were not selected as source or process events but have an impact on resultant events. The results of external impacts calculated by the CIA-ISM model is the critical basis for judging the reasonableness and validity of the established model.

### 2.2. Step 2: Group Estimation Process of Subjective Probabilities

The group estimation process is a time-consuming circular feedback process to estimate the probability of occurrence for each event and conditional probability when other events occur. Experts are required to select relevant events in their specialty fields to determine the subjective probability of occurrence and the conditional probability that reflects the degree of interaction between events. The group estimation process is achieved through the following steps.

(1) Experts are invited to estimate the subjective probability of each event in the identified event set occurring in the studied period. In this paper, the subjective probability values are determined based on the estimation scale shown in Table 1 [4]. It denotes the probability of event i as $P_{i}$.

(2) Experts are invited to estimate a conditional probability that reflect the interactions of selected events. It assumes that if event j occurred, the conditional possibility of event i on event j is denoted as $R_{ij}$. The rules for estimating the conditional possibility are shown in Table 2 [10].

(3) Once the experts have completed their initial estimates, multiple rounds of numerical corrections are required. Then, when a model has been established for the group or an individual, it is possible to vary the initial probabilities of individual events and see the degree of influence that each has on the occurrence of the other events. Experts need to adjust their estimates based on their own experiences and the results of whole group estimates. 

(4) After expert opinion is in agreement, Dalkey’s formula Equation (1) is used to calculate the group estimate for a particular event, rather than using a simple average [18]. The Dalkey method can generate a model having “stronger properties of influence” when a high degree of agreement is reached in the expert estimation [5].

(1) $$GE\left(J/E\right)=\frac{\prod_{i=1}^{n}E_{i}^{J}}{\prod_{i=1}^{n}E_{i}^{J}+\prod_{i=1}^{n}\left(1-E_{i}^{J}\right)}$$

where GE(J/E) denotes the group estimation of event J; n denotes the total number of experts involved in the estimation for event J; $E_{i}^{J}$ denotes the estimation of the ith expert for event J, including the subjective probability of event occurrence and the conditional probability. The calculated group estimation values will be used as the input for the subsequent cross-impact analysis process.

### 2.3. Step 3: Cross-Impact Analysis

The group probability estimates and interaction conditional probability estimates obtained from Step 2 are used as the starting point for the CIA process. This process is essentially a decomposition of the issue and the causalities of the events through the experts’ subjective estimates of event probabilities. The internal cross-impact coefficient matrix C is calculated using the Fermi–Dirac distribution function, as shown in Equation (2) [11].

(2) $$C_{ij}=\frac{1}{1-P_{j}}\left[\ln\left(\frac{R_{ij}}{1-R_{ij}}\right)-\ln\left(\frac{P_{i}}{1-P_{i}}\right)\right]$$

where $C_{ij}$ denotes the internal cross-impact coefficient of event j on event i; $P_{j}$ and $P_{i}$ represent the estimated probability of occurrence of event j and event i, respectively; $R_{ij}$ denotes the estimated conditional possibility of event i on event j.

Once the internal cross-impact coefficients within the event set are calculated, the impacts of external environment on the occurrence probability of the events can be obtained quantitatively. The external impact coefficient was calculated using Equation (3) [6].

(3) $$G_{i}=\ln\left(\frac{P_{i}}{1-P_{i}}\right)-\overset{N}{\underset{k\neq i}{\sum }}C_{ik}P_{k}$$

where $G_{i}$ represents the external impact coefficient of event i; N is the total number of events.

To assess the model’s goodness of fit and explanatory power of the constructed event set, Equation (4) was used to calculate the fitness coefficient I of the model.

(4) $$I=\frac{\sum \left|C_{ij}\right|}{\sum \left|C_{ij}\right|+\sum \left|G_{i}\right|}$$

I indicates the proportion of cross-impact within the event set in the overall event impact. More colloquially, the fitness coefficient I indicates what portion of the probability of occurrence for an event is due to the influence of the event selected in the model. According to the Pareto principle, when I≥80%, the selected set of events is comprehensive and feasible [19].

Once the cross-impact analysis model has been developed, Equation (5) is used to predict the probability of occurrence of one event when the probabilities of other events in the event set change. Notably, Equation (5) is not just an algebraic rearrangement of Equation (3), but an important basis for the derivation of the probability of occurrence in the evolutionary deduction of critical events.

(5) $$P_{i}{}^{\prime }=1/\left[1+\exp\left(-G_{i}-\overset{N}{\underset{k\neq i}{\sum }}C_{ik}P_{k}{}^{\prime }\right)\right]$$

where $P_{k}{}^{\prime }$ refers to the new occurrence probability of event k and $P_{i}{}^{\prime }$ refers to the probability of event i due to the perturbations in other events. $P_{k}{}^{\prime }$ is the updated probability of occurrence in the CIA evolutionary deduction.

### 2.4. Step 4: Graphical Visualization through ISM

Interpretative structural modeling (ISM) is utilized to visualize the interrelationships and causal logic of involved events based on CIA calculation results. ISM forms structured views to effectively detect critical events, which assists the decision makers in making suitable decisions in response to complex emergency situations [20]. The cross-impact coefficient matrix C is treated as the start of ISM modeling, and the process of ISM modeling is described as follows:

#### 2.4.1. Input Matrix Processing

Both positive and negative values are present in cross-impact coefficient matrix C. All the matrix cell values need to be converted to positive values in the ISM model. Matrix C is processed according to the direction of event interaction (i.e., positive or negative) to determine matrix FH. FH matrix and its element $r_{ij}$ are obtained calculated by Equations (6) and (7).


(6) $$r_{ij}=\{\begin{array}{cc} c_{ij}, & event\;j\;has\;a\;positive\;impact\;on\;event\;i\\ 0, & event\;j\;has\;no\;impact\;on\;event\;i\\ -cij, & event\;j\;has\;a\;negative\;impact\;on\;event\;i \end{array}$$

(7) $$FH={\left[r_{ij}\right]}^{T}$$

Positive effects of event i and event j were transformed into elements of (+i,+j) and (−i,−j), and negative effects were transformed into elements of (−i,+j) and (+i,−j), as shown in Table 3 [4]. 

In order to weaken the noise effects of CIA calculation results, we need to select an appropriate cross-impact transformation scale to have the system form stronger causal relationships. The matrix FH is transformed into an adjacency matrix A with Equation (8).

(8) $$A=\left[a_{ij}\right]=\{\begin{array}{cc} 1, & when\;r_{ji}\geq \lambda \\ 0, & when\;r_{ji}<\lambda \end{array}$$

where λ represents the threshold intercept scale to transform FH matrix elements.

Using adjacency matrix A as the input matrix in ISM, the reachability matrix R is calculated by Equation (9) [21].

(9) $${\left(A+I\right)}^{k-1}\neq {\left(A+I\right)}^{k}={\left(A+I\right)}^{k+1}=R$$

where I denotes the unit matrix and k represents the times that the matrix is self-multiplied.

#### 2.4.2. Hierarchical Extraction and Division

For the reachability matrix, three event sets including reachable set R, prior set Q, and common set T are formed. Three sets are classified with the following rules [22]: The reachable set includes all elements corresponding to row values of 1. The prior set consists of all elements corresponding to a column value of 1. The intersection of these two sets is called the common set.

The reachable set and prior set can show an interrelationship between the events but cannot visualize the hierarchy where the events are located. Therefore, cause priority hierarchy extraction from the three element sets is required to establish the event layer [23]. According to the reasoning priority topology hierarchy extraction rule, when $T\left(a_{ij}\right)=Q\left(a_{ij}\right)$, the event is removed and placed at the bottom of the hierarchy. According to the order of sampling, the hierarchical relationship between events can be derived.

#### 2.4.3. Build General Skeleton Matrix and Draw Directed Topological Hierarchy

The core of building a general skeleton is the point and link reduction from the reachability matrix to merge the nodes that have loops and remove duplicate paths. First, the point reduction operation of the reachability matrix R is performed using Tarjan’s SCC algorithm to obtain the reduced point reachability matrix $R^{\prime }$ [24]. The skeleton matrix S is obtained by calculating the reduced reachability matrix $R^{\prime }$ according to Equation (10) [21].

(10) $$S^{\prime }=R^{\prime }-{\left(R^{\prime }-I\right)}^{2}-I$$

where I is the unit matrix. Substituting the circuit elements into $S^{\prime }$ produces the general skeleton matrix S.

The general skeleton matrix obtained by the above operation significantly reduces the complexity of the system. Based on inter-element relationships, a directed topological hierarchy level diagram can be drawn.

## 3. Building COVID-19 Emergency Scenarios with the CIA-ISM Approach

In this section, the process of applying the CIA-ISM approach for building COVID-19 emergency scenarios and constructing a scenario analysis is described.

### 3.1. Event Selection

The occurrence, responses, and post-event impacts of a COVID-19 emergency process were analyzed based on relevant literature [25,26,27,28,29,30]. The identification of the event set considers the existing conditions in the region, dynamic responses from government agencies, and economic-social impacts. After selecting the relevant events, we invited experts in the relevant fields for a collective discussion to judge whether the events selected for this paper are comprehensive and representative. Through literature review and collaborative expert discussions, 17 COVID-19 events were selected for this study, including 7 source events (SE), 7 process events (PE), and 3 resultant events (RE), as shown in Table 4.

### 3.2. Cross-Impact Analysis

#### 3.2.1. Group Estimation and Data Processing

**Figure 2:**
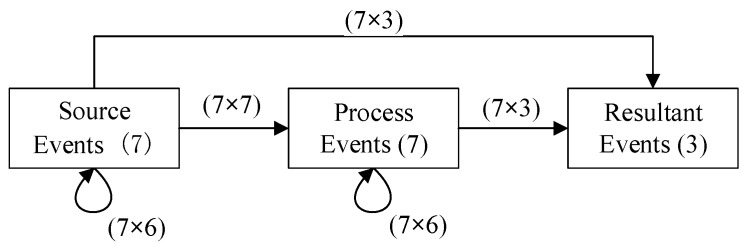
Cross-impact diagram with number of events and number of estimates needed.

Following Step 2 “Group Estimation of Subjective Probabilities” in Section 2, subjective estimates of the possibility of occurrence and the interaction conditional probability of the 17 events were obtained. The expert team needed to make 192 estimates, including 175 interaction conditional probability estimates and 17 probability of occurrence estimates, as shown in Figure 2.

This study was evaluated by three experts from Jiangsu Provincial Health Commission, Jiangsu Provincial Center for Disease Control and Prevention, and Nanjing Municipal Social and Economic Development Research Department in China. Among these experts, there were two people with senior professional titles, and one person with a deputy senior professional title. The expert team is familiar with and has rich research experience with COVID-19 epidemic prevention plans, policies and regulations, the economic impact of the epidemic, and the public psychological impact of the epidemic. Therefore, they were able to provide comprehensive, scientific, and detailed insights into this research.

During the process of group estimation, the expert team carried out three rounds of estimation. In the first round, once the experts finished their individual estimates, we provided the experts with the calculated results of their first estimates. The experts could compare their own estimates with the other estimates and modify them if there were significant deviations. The main purpose of the first round is to avoid problems caused by a biased understanding of concepts such as events, event probability, and event impacts. The second round allowed the experts to discuss and resolve disagreements, including differences in the direction and extent of an event’s impact. After all differences were resolved, the expert group estimation process is complete. In the third round, we applied the ISM to visualize the estimation results [5], and all experts discussed and adjusted the analysis according to visualization of the estimation results.

Table 5 shows the statistics for each round of the expert estimations. Equation (1) was adopted to aggregate the expert group estimates, which produces a more substantial impact attribute when the estimated direction of each unit estimated by the experts has a firm consistency [5]. A decrease in the mean of the internal event impact implies a consensus among experts on the event impact estimate. Table 5 shows an increasing degree of consistency in the experts’ opinions. The final confirmed expert group estimates are shown in Table 6 and Table 7. “OVP” means overall probabilities.

#### 3.2.2. CIA Calculation Process

After obtaining the matrices P (Table 6) and R (Table 7), Equations (2) and (3) were used to calculate the internal cross-impact coefficient matrix C and external impact coefficient vector G for all the events under consideration. The calculation results are shown in Table 8.

The model’s fitness was validated using Equation (4).

(11) $$\left|\mathrm{Internal}\;\mathrm{events}\;\mathrm{impact}\right|=\sum \left|C_{ij}\right|=365.19$$

(12) $$\left|\mathrm{External}\;\mathrm{events}\;\mathrm{impact}\right|=\sum \left|G_{i}\right|=88.71$$

(13) $$\left|\mathrm{Total}\;\mathrm{impact}\right|=\sum \left|C_{ij}\right|+\sum \left|G_{i}\right|=453.90$$

(14) $$\frac{\left|\mathrm{Internal}\;\mathrm{events}\;\mathrm{impact}\right|}{\left|\mathrm{Total}\;\mathrm{impact}\right|}=\frac{\sum \left|C_{ij}\right|}{\sum \left|C_{ij}\right|+\sum \left|G_{i}\right|}=80.46\%$$

The interpretability coefficient of the model constructed in this study reaches more than 80%, which indicates strong reasonableness and explanatory power. This means that the model can reasonably simulate the overall COVID-19 emergency evolution process and identify critical events.

#### 3.2.3. Resultant Events Analysis

The data for the resultant event analysis were obtained from the rows corresponding to RE1, RE2 and RE3 (i.e., the affected degree of RE1, RE2 and RE3) in Table 9. We sorted the row data for RE1, RE2 and RE3 and analyzed them according to their numerical magnitude ranking, as shown in Table 9, Table 10 and Table 11.

The internal cross-influences of source events and process events are used as an objective numerical judgment basis for analyzing the resultant events. As shown in Table 9, sufficient medical resources (event SE4) and a sound urban epidemic prevention and emergency command system (event SE6) are at the core of effective control of a COVID-19 outbreak. Epidemics occurring in cities with peak population movements (event SE1) and transportation centers (event SE2) are not conducive for effective control of the epidemic.

The government’s epidemic prevention publicity (event SE3) can effectively reduce the possibility of social panic caused by an epidemic. Besides the timing and location of the spread of the epidemic, the timeliness of the release of the government’s epidemic information (event PE1) can have an enormous impact on social sentiment, as shown in Table 10.

As seen in Table 11, the outbreak of epidemics in transportation-centric cities (event SE2) is often a precursor to enormous social and economic losses (RE2). If the government can effectively isolate infected people and their close contacts (event SE3) and fully complete the epidemiological investigation (event PE2), social and economic losses can be minimized.

### 3.3. Scenario Analysis

#### 3.3.1. Scenario Generation and Evolutionary Analysis 

**Figure 3:**
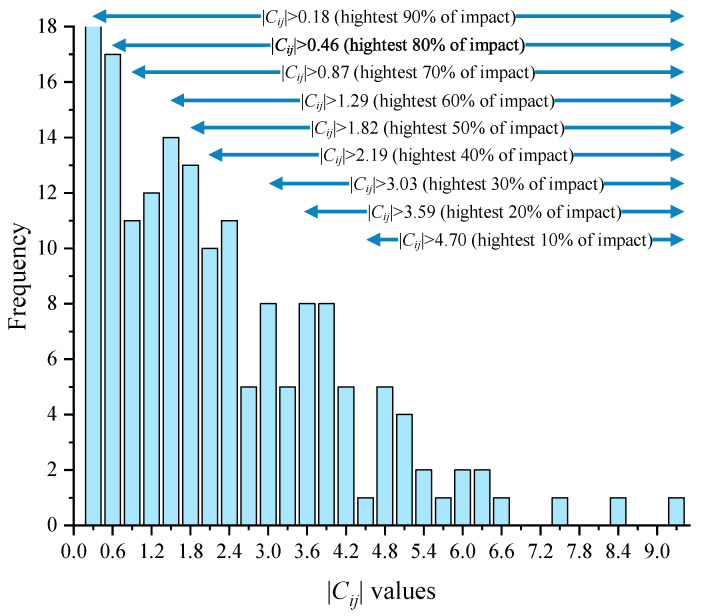
Histogram of the $\left|C_{ij}\right|$ distribution.

After the internal cross-impact coefficient matrix C was calculated, ISM was used to draw the directed topology hierarchy based on the Step 4 “Graphical Visualization Through ISM” in Section 2. The scenario directed graph generated by ISM can show the direction and degree of impact between events. By choosing different intercepts to observe changes in the logic of event occurrence, we can deepen our understanding of the degree of mutual influence between events and the links between events. This process aims to select an appropriate $\left|C_{ij}\right|$ value as the threshold intercept for generating the scenario. The distribution of $\left|C_{ij}\right|$ is plotted as a histogram in Figure 3. If $\left|C_{ij}\right|>4.70$ is selected as the threshold intercept for ISM modeling, the ISM directed topological hierarchy diagram will contain the highest 10% of internal cross-impacts. The COVID-19 emergency management scenario maps generated by ISM can reflect the direction and degree of impacts between selected events.

**Figure 4:**
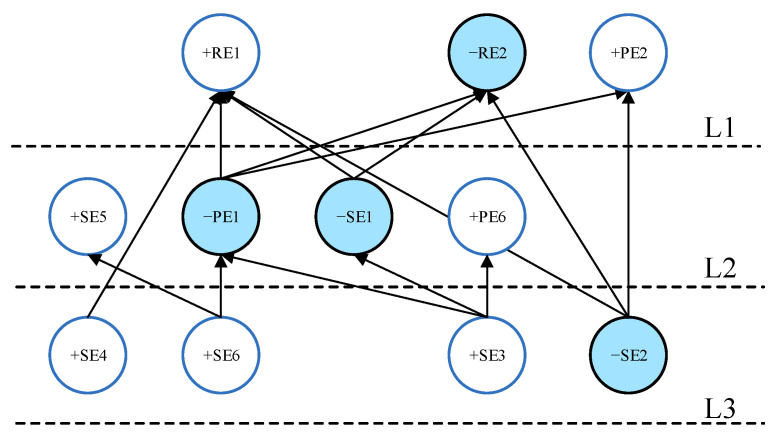
Digraph for the limit $\left|C_{ij}\right|>4.70$.

Figure 4 shows the structure diagram of the COVID-19 outbreak scenario obtained when $\left|C_{ij}\right|>4.70$ is selected as the threshold. Notably, the structure diagram in Figure 4 includes only 11 of the 17 events in the event set. Events SE7, PE3, PE4, PE5, PE7, and RE3 do not appear. This means that when the event’s impact is limited to the top 10%, RE3 (reducing socio-economic losses) is not the most critical consequence, whereas RE1 (effectively controlling the epidemic) and RE2 (effectively preventing social panic) are the top priorities for the entire epidemic prevention and control. This phenomenon is in accordance with the current community-based dynamic zeroing prevention policy in mainland China. The community-based dynamic zeroing policy has played a pivotal role in minimizing the number of laboratory-confirmed cases and deaths, but it caused some degree of socio-economic loss in mainland China [31]. When there is a sudden outbreak of COVID-19, this policy puts the safety of people’s lives and the maintenance of social stability in first place, which is the only way to have a better economic recovery after a disaster. The location of the COVID-19 outbreak (event SE2), public awareness and education on epidemic prevention (event SE3), an adequate reserve of emergency medical resources (event SE4), and a sound urban epidemic prevention and emergency command system (event SE6) are the underlying primary events in the prevention and control of a COVID-19 outbreak. The most direct and critical events for preventing social panic are that the epidemic does not occur during the peak period of population movement (event −SE1) and the government releases COVID-19 outbreak information on time (event −PE1). Besides the event (−SE1), adequate reserves of emergency supplies (event +SE4) and the government release of COVID-19 outbreak information on time (event −PE1) are the core events that can effectively control a COVID-19 epidemic.

After a comprehensive understanding of the event hierarchy in the above scenario, we can perform an evolutionary analysis. In this scenario evolution analysis, we set the probabilities of occurrence for the underlying events SE3 and SE7 to 0 and the probabilities of occurrence for SE4 and PE1 to 1. The probabilities of occurrence for other events are then calculated using Equation (5). If one event has a probability of occurrence greater than 0.99 or smaller than 0.01, then it is considered certain to occur or disappear, respectively. This looping process continues until all the selected events in the event set are sure to have occurred or not. Table 12 shows the event evolution process.

Based on the evolution results, it is noted that only having sufficient emergency supplies (SE4) will not be able to effectively control the epidemic (+RE1) if a complete collaborative governance system is not formed (+SE7) and the importance of publicity is ignored for epidemic prevention (−SE3). Eventually, social panic (+RE2) can occur, but not necessarily for the socio-economy (+RE3). This COVID-19 emergency scenario evolution analysis can provide stakeholders with a clear and comprehensive understanding of the causality structure of the events in Figure 4. 

#### 3.3.2. Sensitivity Analysis

In order to understand the causal connections between the selected events, we need to analyze the system structure scenarios at different levels of impact by sensitivity analysis. In addition to analyzing the situation when $\left|C_{ij}\right|>4.70$, we selected 20%, 30% and 40% impacts in the C distribution as thresholds to analyze the event structure in order to predict possible COVID emergency scenarios. 

**Figure 5:**
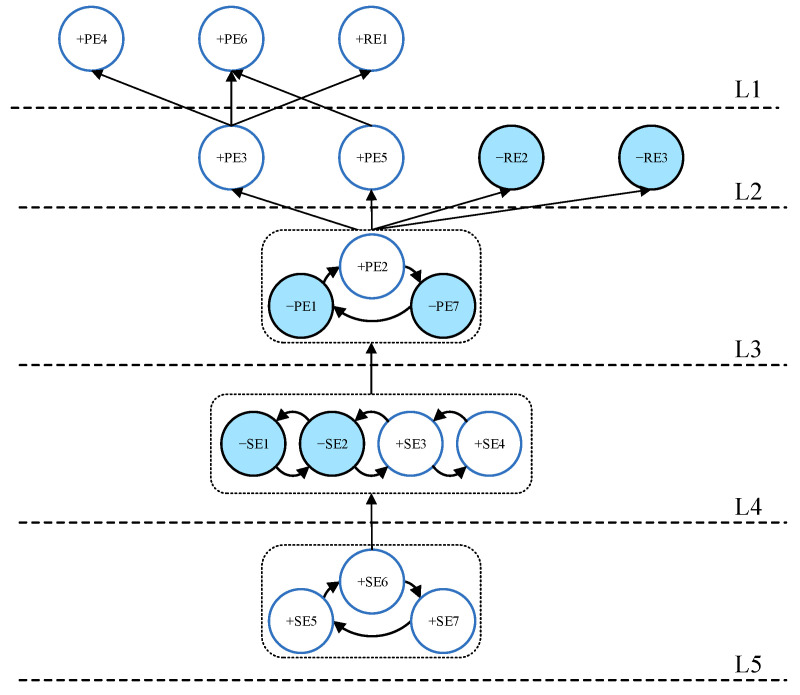
Digraph for the limit $\left|C_{ij}\right|>2.19$.

Figure 5 shows the event hierarchy for the highest 40% impact scenario. As can be seen in Figure 5, (loop events “+SE5, +SE6, +SE7”), (loop events “−PE1, +PE2, −PE7”), and (loop events “−SE1, −SE2, +SE3, +SE4”) make up three micro-scenarios. This means that these events have significant influences on each other and generally happen together. In addition, the emergence of micro-scenarios can provide insights for outbreak managers. For example, establishing a multi-channel early warning mechanism, an effective urban emergency command system, and a regional collaborative governance system (loop events “+SE5, +SE6, +SE7”) are the most fundamental initiatives for controlling the epidemic. Therefore, more attention could be given to strengthening cooperation with the relevant departments in this micro-scenario.

**Figure 6:**
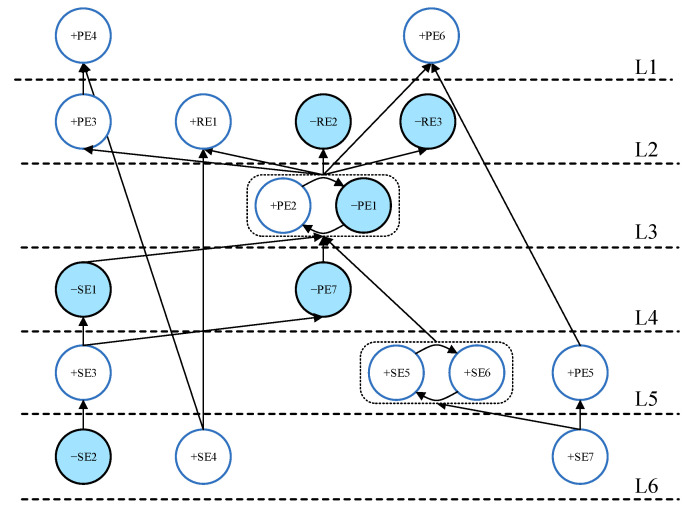
Digraph for the limit $\left|C_{ij}\right|>3.03$.

Compared with the scenario in Figure 5, the top 30% impact structure graph (Figure 6) shows apparent hierarchical layers, which can be used to understand the logical causal relationships between events. The micro-scenario (loop events “+SE5, +SE6, +SE7”) in layer L5 in Figure 5 is split into a micro-scenario (loop events “+SE5, +SE6”) and one single event (+SE7) in Figure 6. Based on this split relationship and combined with the event hierarchy, we can predict that establishing an effective regional collaborative governance system (+SE7) is necessary to achieve multi-channel detection and warning and an effective epidemic emergency command system (loop events “+SE5, +SE6”). The event (+SE7) and the event (+SE4) are located at the bottom of the ISM hierarchy, which means that a collaborative governance system and sufficient reserves of medical emergency resources are the most fundamental events in COVID-19 outbreak emergency management. The timely release of government information (−PE1) and epidemiological investigation and traceability (+PE2) are the most directly influential factors that effectively control the outbreak and reduce social panic and economic losses.

**Figure 7:**
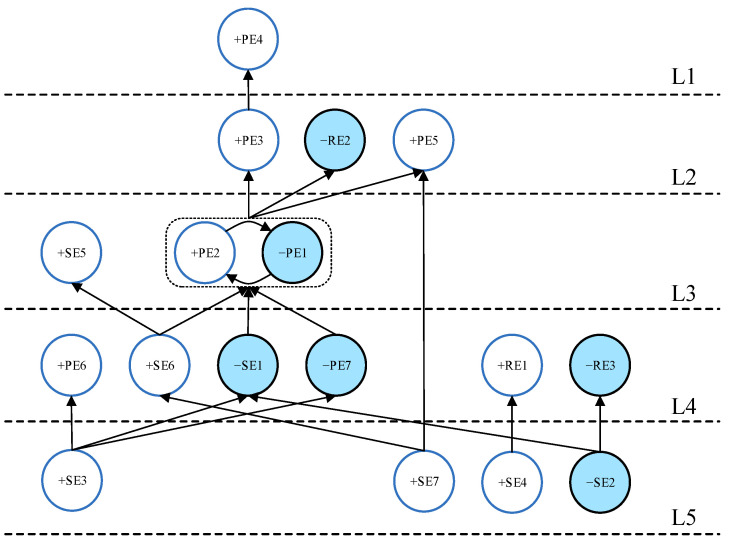
Digraph for the limit $\left|C_{ij}\right|>3.72$.

The top 20% impact structure shown in Figure 7 is more optimized than in Figure 6. Only one micro-scenario (loop events “−PE1, +PE2”) remains in Figure 7. The completion of government epidemiological investigation and traceability (event +PE2) and the timely release of government information (event −PE1) often occur simultaneously. The interaction between event −PE1 and +PE2 is the strongest among all of the process events. This means that PE1 and PE2 can be combined as one process event for outbreak prevention and control.

We can visually analyze the most critical events or event chains regarding the occurrence of the resultant events based on Figure 7. Thus, sufficient reserves of medical emergency resources (event +SE4) are essential for effectively controlling the spread of the COVID-19 epidemic (event +RE1). The key to preventing the major social and economic losses caused by the epidemic (event RE3) is to prevent outbreaks in transportation-centric cities (event −SE2). Outbreaks that do not occur at the peak of population movement (−SE1) often play a positive role for the timely publication of outbreak information and epidemiological investigations by the government (loop events “−PE1, +PE2”). A city with a well-developed emergency command system (+SE6) and the ability of government officials to implement preventive and control measures following regulations (−PE7) are necessary to achieve the timely publication of outbreak information and adequate epidemiological investigation (loop events “−PE1, +PE2”). Combining the “Resultant Events Analysis” in Section 3 and Figure 7, we can analyze that (event +SE3)→(event −PE7)→(loop events “−PE1, +PE2”)→(event −RE2) is the critical path for controlling social panic caused by the outbreak (event −RE2). To effectively control social panic during an epidemic, the public needs to be adequately educated about the outbreak before it occurs (event +SE3) so that supervisors and managers can effectively implement outbreak prevention policies that follow regulations (event −PE7). On this basis, timely publication of epidemic-related information and adequate epidemiological investigation by the government (loop events “−PE1, +PE2”) can effectively reduce the occurrence of social panic (event −RE2).

Scenario sensitivity analysis helps managers understand the specific impact paths and underlying logic that leads to resultant events. Scenario evolution analysis can predict the dynamic evolution process of events related to outbreak prevention and control management. The resultant event analysis can be used as a complement to the scenario sensitivity analysis and evolution analysis to represent the static direct impact of source and dynamic events on the resultant events. By combining the results of critical scenario generation, scenario evolution analysis, scenario sensitivity analysis, and resultant event analysis, we can comprehensively and clearly understand the logical relationships among the involved events and their essential roles in COVID-19 prevention and control.

## 4. Case Study and Results

In this section, the prevention and control of the COVID-19 emergency in Nanjing in 2021 is used as a case scenario to demonstrate the feasibility of using our CIA-ISM approach for evaluating the effectiveness of COVID-19 emergency management.

### 4.1. COVID-19 Emergency Conditions in Nanjing

On 20 July 2021, the Nanjing epidemic began when nine new coronavirus cases were detected by routine nucleic acid tests at Nanjing Lukou International Airport. Nanjing, in the Yangtze River region, is one of the biggest cities in China with a population of 942.34 million and plays a critical hub role in national transportation. As the capital city of Jiangsu Province, Nanjing owns rich medical emergency supplies and resources, has a sound emergency regulation and command system, and is directly capable of epidemic prevention and publicity work. The total COVID-19 vaccination rate for the resident population has reached 88.8% in the Jiangsu Province. As a megacity, Nanjing also has a comprehensive governance collaboration system. However, since the outbreak of COVID-19 started in Nanjing’s airport, it has been treated as an important place for epidemic prevention, but some deficiencies might exist in Nanjing’s multi-channel monitoring and early warning mechanism. Based on the above analysis, Table 13 summarizes the conditions of COVID-19 emergency source events for Nanjing city.

The occurrence timeline of COVID-19 emergency process events during the Nanjing epidemic is shown in Table 14. The occurrence probabilities for the process events that certainly occur are set to 1. The process events that are uncertain are assumed to have a probability of 0.5. Through the analysis of Table 14, we used six steps to simulate the emergency management process during the 20 days after the COVID-19 outbreak in Nanjing. All event settings for each stage are shown in Table 15.

### 4.2. Deduction Results Analysis

**Figure 8:**
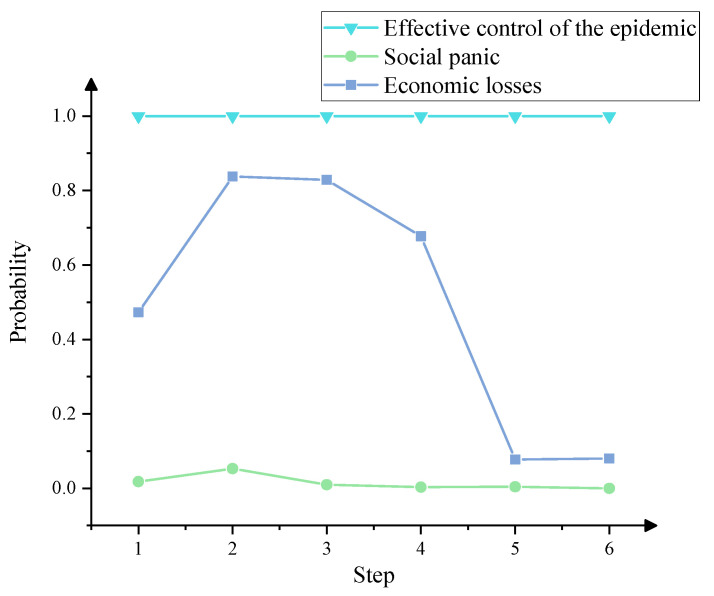
Prediction probabilities trend chart of resultant events.

We performed scenario deduction analysis according to the condition settings summarized in Table 15. By applying Equation (5), the new occurrence probabilities of other events in each step of the scenario can be predicted, as shown in Table 16. The variation trend for the occurrence probabilities of the three resultant events is shown in Figure 8.

**Figure 9:**
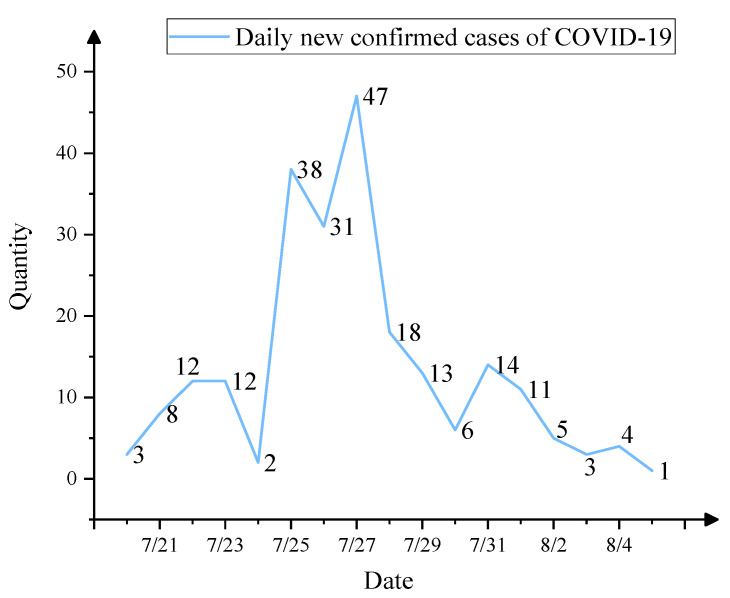
Daily new confirmed cases of COVID-19 from 20 July to 5 August.

In order to verify the feasibility of applying the CIA-ISM approach, this paper compared simulated emergency evolution scenarios with actual events in the Nanjing epidemic. Intensive virus outbreaks occurred in residential communities near Nanjing Lukou International Airport, with a high percentage of infected populations. The number of new confirmed cases in the Nanjing outbreak peaked on 27 July 2021, and then gradually declined, as shown in Figure 9 [32]. Moreover, no severe or fatal cases were reported among locally confirmed cases in Nanjing. Our prediction (Figure 8) shows that the probability of effective control of the epidemic had been maintained at a level close to 1, and the overall situation in Nanjing was controllable. The evolutionary scenario predicted by CIA-ISM showed significant consistency with the actual COVID-19 situation in Nanjing. Effective control of the epidemic was mainly due to Nanjing’s complete emergency command system and collaborative governance system and outbreak prevention education. According to the Sina Weibo Hotspot Ranking during the epidemic, the degree of public concern about the Nanjing epidemicwas relatively high, and it peaked on 25 July. There had been a series of public concerns and attentions related to the Nanjing epidemic, such as the spreading trend of the Nanjing epidemic, Nanjing Lukou Airport operation, medical treatment situation during the Nanjing epidemic, and so on. In the subsequent period, the popularity of public concern gradually decreased. This is consistent with what we calculated: the possibility of social panic first increased and then decreased. The public opinion crisis was avoided mainly because the Nanjing government could effectively monitor public attention, release information about the epidemic on time, and do an excellent job of pacifying the emergency. The probability of significant economic loss predicted by CIA-ISM fluctuates wildly, with a peak of 0.8376 (Figure 8). This means a greater likelihood of severe financial loss during the Step 2–4 scenarios. These scenarios represent the same periods when many high-risk areas in Nanjing shut down their production. 

## 5. Discussion

### 5.1. CIA-ISM Method Applications and Its Intrinsic Logic

The CIA method integrates probability with interaction factors in calculating internal cross-impact coefficients [11]. Compared to Bayesian network analysis, CIA does not require prior knowledge of the structure of process evolution, which reduces the amount of preparation before analysis and the number of estimates needed to identify evolutionary paths [4,11]. In addition, the CIA method transforms the probability or degree of impact of the calculated results from a non-linear representation (0, 1) of the interval to a linear representation (−:, +:) of the interval [4]. This allows managers to recognize positive versus negative impact relationships between two events. Internal cross-impact coefficients are solved by n independent equations in the CIA approach, each solution providing a single result, which allows experts to see their own model outcomes prior to group estimate processing, providing a more intuitive basis for expert group collaboration [4]. The high flexibility and integrability of the CIA method make it possible to produce better results in combination with other visual analysis methods, such as ISM, meta-network analysis (MNA), and functional resonance analysis model (FRAM). The ISM method allows scene generation and visualization of CIA calculation results [10]. It converts the CIA calculation results into a directed topology hierarchy graph. The directedness of the links demonstrates the causal association of sub-events in the source events, process events, and resultant events [5,6]. The ISM hierarchical structure adopts the principle of cause-priority hierarchy and places the cause-type events in the lower layer [4] to help us understand the causal properties of events comprehensively [20]. In addition, we can select appropriate thresholds to demonstrate different event relationship structures based on planned goals [6,10]. With different thresholds, events will exhibit different micro-scenarios, macro-scenarios, and evolutionary trends and we can capture the cascading effects between events when emergencies arise. Furthermore, before ISM scenario generation, these causal emergent features are unknown to us. ISM enables the structured and causal presentation of the unordered results of CIA [4]. 

### 5.2. Developing Scenario-Based Emergency Management for COVID-19 Outbreak Prevention and Control

Based on the results for critical event identification and cause-effect correlation analysis, the core of establishing an effective emergency management for epidemic prevention and control in a COVID-19 crisis is to develop a regional collaborative governance system (event SE7). Regional collaborative governance includes effective crisis prevention and reconstruction of public health through collaborative implementation of cross-regional resource allocation, information sharing, and mutual funds assistance [33,34]. First, it is necessary to realize a regional collaborative governance system to dispatch medical supplies, healthcare facilities, and medical personnel for epidemic prevention and control in a comprehensive manner (event SE4). By establishing a regional collaborative governance system, the fragmented allocation of emergency resources can be avoided. Second, strengthening inter-regional information communication can improve multi-channel epidemic detection and early warning capabilities (event SE5). The government’s integrated emergency command system (event SE6) should be improved at different stages (prevention, resistance, and recovery). In addition, publicity and education on public epidemic prevention (event SE3) should be strengthened to create social, cultural, and legal systems where people can actively participate in outbreak emergency management. Combining epidemic prevention and education with collaborative regional governance based on epidemic scenarios to improve the government’s early warning capability for public health emergencies can reduce the destructive power of epidemics and rapidly restore economic and social stability.

## 6. Conclusions

This paper introduced a CIA-ISM based model for assessing the effectiveness of COVID-19 outbreak emergency management. This paper firstly developed the epidemic emergency management event set, including source events, process events, and resultant events. Experts in public health management fields were invited to estimate the subjective probabilities of all events and the interaction impacts between events. CIA-ISM is used to calculate expert group estimation for scenario generation with different thresholds for COVID-19 emergency management, critical impact event analysis, and evaluation of the effectiveness of management measures based on scenario evolution analysis. In order to verify the feasibility and applicability of the established COVID-19 emergency management scenario model based on CIA-ISM, the model was applied to a simulation of the COVID-19 outbreak in Nanjing on 20 July 2021. The evolution prediction results of the proposed model were consistent with the development of the Nanjing COVID-19 epidemic and had forward-looking capabilities. 

We analyzed the results of critical event identification, internal cross-impacts, scenario generation, and evolutionary scenario deduction. Public education on epidemic prevention, medical supply reserves, and a collaborative regional governance system were the most critical factors for effectively controlling the epidemic and alleviating social panic. Among the top 10–40% most significant impacts scenarios, the micro-scenarios consisting of epidemiological investigation and traceability and adequate, timely nucleic acid testing of critical populations were the most strongly interacting loops. Finally, we made specific recommendations for COVID-19 outbreak management to improve future emergency management.

This paper is the first to apply the CIA-ISM method to the field of COVID-19 emergency management, broadening the application scenario of the method. One of the most critical features of the method is its ability to integrate expertise from multiple related fields into COVID-19 emergency management. This paper extends the research perspective of COVID-19 emergency management by focusing on a scenario-based quantitative analysis of the beginning, development, and results of outbreak emergency management. In addition, the COVID-19 emergency management CIA-ISM model developed in this paper achieves critical event identification, scenario causation presentation, and dynamic scenario evolution, providing managers with a comprehensive, multi-level awareness and understanding of COVID-19 emergency management.

There are some limitations in this paper. The group estimation data in this paper was provided by three experts in relevant fields, and the expert panel was small but met the minimum size required by the literature [4,5]. A somewhat larger, more diverse group of experts would add more valuable information. Notwithstanding, from our perspective these limitations do not have a critical impact on the validity of the results due to the coherency of the outcomes obtained in the simulation. In addition, most of this paper selected macro events related to outbreak emergency management and did not select COVID-19 virus attributes as source events. In the case study section of this paper, the most intuitive reality was chosen as a realistic control for the predicted outcome of the resultant event, rather than the most accurate reality. This issue has no significant impact on the effectiveness of the COVID-19 emergency management CIA-ISM model. Future research should focus on extending the model to analyze the effectiveness of epidemic emergency management in different settings in other countries to verify the general validity of the model. Future studies could improve the predictive accuracy of the model by refining the selection of COVID-19 prevention and control events, such as the transmissibility, virulence, and mode of transmission of the virus. In addition, we could merge the micro-scenarios in the scenarios and expand the selected events in terms of the social impacts caused by the epidemic and government emergency management. Finally, the validity of the model could be increased by expanding the size of the expert panel and the areas covered by the experts to obtain more comprehensive and valuable information.

## Figures and Tables

**Table 1:** Subjective probability estimation scale [4].

Description	Possibility (%)	Description	Possibility (%)
Very unlikely	5	Possible	60
Highly unlikely	15	Likely	75
Unlikely	25	Highly likely	85
Possibly not	40	Almost certain	95
Uncertain	50		

**Table 2:** Conditional probability estimation scale.

Conditional Possibility Estimation Value	Explanation
0.99	j has a significant positive impact on i.
0.9	j has an obvious positive impact on i.
0.8	j has a great positive impact on i.
0.7	j has a certain positive impact on i.
0.6	j has a slight positive impact on i.
0.5	j has no impact on i.
0.4	j has a slight negative impact on i.
0.3	j has a certain negative impact on i.
0.2	j has a great negative impact on i.
0.1	j has an obvious negative impact on i.
0.01	j has a significant negative impact on i.

**Table 3:** The matrix transformation table.

	Event Occurrence (+i)	Event Non-Occurrence (−i)
Event occurrence (+i)	$c_{ij}$	$-c_{ij}$
Event non-occurrence (−i)	$-c_{ij}$	$c_{ij}$

**Table 4:** Critical events of COVID-19 prevention and control for the CIA-ISM model.

Event ID	Event	Source
SE1	The spread of COVID-19 occurs at the peak of population movements.	[25,29]
SE2	The COVID-19 outbreak is in a transportation hub city.	[25,29]
SE3	The government’s COVID-19 epidemic prevention education and training are in place.	[26,28]
SE4	The city has sufficient reserves of medical emergency resources.	[26,28,29]
SE5	The multi-channel epidemic monitoring and forewarning mechanism is established.	[26,27,29]
SE6	The urban epidemic prevention and emergency command systems are sound.	[26,27,29]
SE7	The urban collaborative governance system is established.	[25,27,29]
PE1	The government does not release COVID-19 outbreak information on time.	[26,29,30]
PE2	Epidemiological surveying and tracking is completed in a timely manner.	[25,26,29]
PE3	The government has effectively completed the isolation of infected people and their close contacts.	[25,26,28,29]
PE4	Nucleic acid testing of critical populations is timely.	[25,26,28,29]
PE5	Supplies transported from other regions can be delivered on time.	[25,29]
PE6	The government can effectively channel public opinions and address public discontent.	[28,30]
PE7	The law-based prevention and control measures are not in place.	[27,28,30]
RE1	The COVID-19 epidemic has been effectively controlled, and no large-scale infection has occurred.	[26,28,29]
RE2	Ineffective COVID-19 epidemic prevention has caused public grievances and social panic.	[28,30]
RE3	The COVID-19 epidemic has caused enormous social and economic losses.	[26,27,29]

**Table 5:** Group estimation statistics table.

Round	Conflicts	Internal Event Impact $\sum \left|C_{ij}\right|$	External Event Impact $\sum \left|G_{i}\right|$	Internal Event Impact Mean	Fitness Coefficient I
1	38	1226.98	828.54	7.01	59.68%
2	19	499.40	253.07	2.85	66.37%
3	0	365.19	88.71	2.11	80.57%

**Table 6:** Estimated probability of event occurrence matrix P.

SE1	SE2	SE3	SE4	SE5	SE6	SE7	PE1	PE2	PE3	PE4	PE5	PE6	PE7	RE1	RE2	RE3
0.53	0.60	0.80	0.65	0.50	0.55	0.45	0.48	0.70	0.65	0.55	0.50	0.55	0.35	0.78	0.25	0.35

**Table 7:** Estimated conditional probability matrix R.

R	SE1	SE2	SE3	SE4	SE5	SE6	SE7	PE1	PE2	PE3	PE4	PE5	PE6	PE7
SE1	OVP	0.83	0.30	0.50	0.20	0.43	0.43	0.00	0.00	0.00	0.00	0.00	0.00	0.00
SE2	0.77	OVP	0.73	0.57	0.43	0.43	0.43	0.00	0.00	0.00	0.00	0.00	0.00	0.00
SE3	0.50	0.50	OVP	0.53	0.50	0.70	0.63	0.00	0.00	0.00	0.00	0.00	0.00	0.00
SE4	0.37	0.42	0.70	OVP	0.50	0.80	0.63	0.00	0.00	0.00	0.00	0.00	0.00	0.00
SE5	0.50	0.50	0.50	0.50	OVP	0.90	0.73	0.00	0.00	0.00	0.00	0.00	0.00	0.00
SE6	0.50	0.50	0.50	0.57	0.87	OVP	0.90	0.00	0.00	0.00	0.00	0.00	0.00	0.00
SE7	0.57	0.43	0.50	0.50	0.77	0.70	OVP	0.00	0.00	0.00	0.00	0.00	0.00	0.00
PE1	0.83	0.73	0.20	0.43	0.20	0.10	0.33	OVP	0.23	0.47	0.47	0.50	0.40	0.73
PE2	0.23	0.23	0.63	0.67	0.73	0.67	0.87	0.10	OVP	0.60	0.60	0.50	0.70	0.17
PE3	0.23	0.23	0.57	0.80	0.67	0.80	0.73	0.47	0.87	OVP	0.73	0.67	0.67	0.33
PE4	0.20	0.20	0.70	0.80	0.70	0.87	0.73	0.13	0.83	0.83	OVP	0.67	0.70	0.30
PE5	0.50	0.73	0.60	0.67	0.63	0.73	0.90	0.37	0.70	0.50	0.73	OVP	0.70	0.30
PE6	0.50	0.23	0.87	0.73	0.70	0.30	0.73	0.20	0.70	0.73	0.77	0.87	OVP	0.17
PE7	0.67	0.53	0.20	0.33	0.30	0.17	0.30	0.70	0.23	0.23	0.33	0.27	0.33	OVP
RE1	0.17	0.23	0.77	0.99	0.80	0.99	0.90	0.20	0.80	0.90	0.80	0.83	0.77	0.27
RE2	0.80	0.70	0.10	0.20	0.27	0.17	0.10	0.87	0.27	0.17	0.20	0.23	0.17	0.87
RE3	0.67	0.77	0.37	0.33	0.33	0.27	0.20	0.57	0.17	0.23	0.30	0.30	0.27	0.67

**Table 8:** Cross-impact matrix.

C	SE1	SE2	SE3	SE4	SE5	SE6	SE7	PE1	PE2	PE3	PE4	PE5	PE6	PE7	RE1	RE2	RE3
SE1	OVP	3.63	−4.90	−0.38	−3.04	−0.89	−0.73	0.00	0.00	0.00	0.00	0.00	0.00	0.00	0.00	0.00	0.00
SE2	1.72	OVP	3.03	−0.35	−1.35	−1.50	−1.22	0.00	0.00	0.00	0.00	0.00	0.00	0.00	0.00	0.00	0.00
SE3	−2.97	−3.47	OVP	−3.62	−2.77	−1.20	−1.53	0.00	0.00	0.00	0.00	0.00	0.00	0.00	0.00	0.00	0.00
SE4	−2.47	−2.35	1.14	OVP	−1.24	1.71	−0.13	0.00	0.00	0.00	0.00	0.00	0.00	0.00	0.00	0.00	0.00
SE5	0.00	0.00	0.00	0.00	OVP	4.88	1.81	0.00	0.00	0.00	0.00	0.00	0.00	0.00	0.00	0.00	0.00
SE6	−0.43	−0.50	−1.00	0.19	3.40	OVP	3.63	0.00	0.00	0.00	0.00	0.00	0.00	0.00	0.00	0.00	0.00
SE7	1.03	−0.17	1.00	0.57	2.82	2.33	OVP	0.00	0.00	0.00	0.00	0.00	0.00	0.00	0.00	0.00	0.00
PE1	3.59	2.70	−6.60	−0.58	−2.64	−4.73	−1.14	OVP	−3.74	−0.19	−0.15	0.13	−0.75	1.63	0.00	0.00	0.00
PE2	−4.36	−5.09	−1.50	−0.40	0.33	−0.34	1.92	−5.82	OVP	−1.26	−0.98	−1.69	0.00	−3.78	0.00	0.00	0.00
PE3	−3.88	−4.52	−1.75	2.19	0.15	1.71	0.71	−1.46	4.18	OVP	0.83	0.15	0.16	−2.02	0.00	0.00	0.00
PE4	−3.40	−3.97	3.23	3.39	1.29	3.78	1.47	−4.01	4.70	4.03	OVP	0.98	1.44	−1.61	0.00	0.00	0.00
PE5	0.00	2.53	2.03	1.98	1.09	2.25	3.99	−1.06	2.82	0.00	2.25	OVP	1.88	−1.30	0.00	0.00	0.00
PE6	−0.43	−3.52	8.36	2.32	1.29	−2.33	1.47	−3.07	2.16	2.32	2.20	3.40	OVP	−2.75	0.00	0.00	0.00
PE7	2.84	1.88	−3.84	−0.21	−0.46	−2.15	−0.42	2.84	−1.90	−1.63	−0.16	−0.79	−0.16	OVP	0.00	0.00	0.00
RE1	−6.20	−6.23	−0.48	9.46	0.20	7.36	1.66	−5.17	0.34	2.61	0.22	0.60	−0.21	−3.53	OVP	0.00	0.00
RE2	5.32	4.86	−5.49	−0.82	0.17	−1.08	−2.00	5.81	0.29	−1.46	−0.64	−0.18	−1.08	4.61	0.00	OVP	0.00
RE3	2.84	4.57	0.36	−0.21	−0.15	−0.87	−1.40	1.74	−3.22	−1.63	−0.51	−0.46	−0.83	2.02	0.00	0.00	OVP
G	4.47	−0.66	10.13	2.18	−3.50	−1.93	−4.52	9.09	13.20	1.91	−8.46	−11.29	−9.15	3.13	−0.82	−3.16	−1.12

**Table 9:** Cross-impact ranking of source and process events on resultant event RE1.

Event ID	SE4	SE6	PE3	SE7	PE5	PE2	PE4	SE5	PE6	SE3	PE7	PE1	SE1	SE2
$C_{ij}$	9.46	7.36	2.61	1.66	0.60	0.34	0.22	0.20	−0.21	−0.48	−3.53	−5.17	−6.20	−6.23

**Table 10:** Cross-impact ranking of source and process events on resultant event RE2.

Event ID	PE1	SE1	SE2	PE7	PE2	SE5	PE5	PE4	SE4	PE6	SE6	PE3	SE7	SE3
$C_{ij}$	5.81	5.32	4.86	4.61	0.29	0.17	−0.18	−0.64	−0.82	−1.08	−1.08	−1.46	−2.00	−5.49

**Table 11:** Cross-impact ranking of source and process events on resultant event RE3.

Event ID	SE2	SE1	PE7	PE1	SE3	SE5	SE4	PE5	PE4	PE6	SE6	SE7	PE3	PE2
$C_{ij}$	4.57	2.84	2.02	1.74	0.36	−0.15	−0.21	−0.46	−0.51	−0.83	−0.87	−1.40	−1.63	−3.22

**Table 12:** Simulation probability for non-occurrence of SE3 and SE7, and occurrence of SE4 and PE1.

	Step1	Step2	Step3	Step4	Step5	Step6	Step7	Step8	Step9	Step10
SE1	0.53	0.98	0.72	1.00	1.00	1.00	1.00	1.00	1.00	1.00
SE2	0.60	0.07	0.07	0.03	0.05	0.05	0.00	0.00	0.00	0.00
SE3	0.00	0.00	0.00	0.00	0.00	0.00	0.00	0.00	0.00	0.00
SE4	0.00	0.00	0.00	0.00	0.00	0.00	0.00	0.00	0.00	0.00
SE5	0.50	0.73	0.95	0.96	0.96	1.00	1.00	1.00	1.00	1.00
SE6	0.55	0.95	0.98	1.00	1.00	1.00	1.00	1.00	1.00	1.00
SE7	1.00	1.00	1.00	1.00	1.00	1.00	1.00	1.00	1.00	1.00
PE1	1.00	1.00	1.00	1.00	1.00	1.00	1.00	1.00	1.00	1.00
PE2	0.70	0.59	0.45	0.84	0.62	0.52	0.69	1.00	1.00	1.00
PE3	0.65	0.56	0.33	0.44	0.63	0.37	0.41	0.41	1.00	1.00
PE4	0.55	0.00	0.00	0.00	0.00	0.00	0.00	0.00	0.00	0.00
PE5	0.50	0.22	0.00	0.00	0.00	0.00	0.00	0.00	0.00	0.00
PE6	0.55	0.00	0.00	0.00	0.00	0.00	0.00	0.00	0.00	0.00
PE7	0.35	0.98	0.98	0.98	1.00	1.00	1.00	1.00	1.00	1.00
RE1	0.78	0.00	0.00	0.00	0.00	0.00	0.00	0.00	0.00	0.00
RE2	0.25	1.00	1.00	1.00	1.00	1.00	1.00	1.00	1.00	1.00
RE3	0.35	0.35	0.61	0.64	0.44	0.57	0.66	0.66	0.31	1.00

**Table 13:** Source events setting for the Nanjing COVID-19 epidemic.

Event ID	SE1	SE2	SE3	SE4	SE5	SE6	SE7
Probability	0	1	1	1	0	1	1

**Table 14:** The process events timeline for the Nanjing COVID-19 epidemic.

Time	Event
21 July	Nanjing held a press conference to inform Nanjing Lukou International Airport of the epidemic situation.
22 July	Nanjing conducted the first round of full nucleic acid testing. The Nanjing government effectively transferred and isolated infected people and their close contacts. Cases of Nanjing-associated infectious diseases were found in Anhui, Liaoning, and Guangdong provinces.
24 July	The emergence of a new pattern of inter-province spread of the epidemic in Nanjing has triggered a new pattern of domestic spread and public anxiety.
25 July	Nanjing conducted the second round of full nucleic acid testing.
28 July	Nanjing conducted the third round of full nucleic acid testing. Nanjing’s public opinion appeasement work was carried out to avoid the occurrence of a public opinion crisis.
29 July	The epidemic spread from Nanjing to 19 cities, with a trend of polycentric spread.
30 July	According to the press conference on epidemic prevention and control in Nanjing, the source of the current epidemic in Nanjing was the inbound flight CA910 from Russia due to Delta virus strain.
2 August	Nanjing conducted the fourth round of full nucleic acid testing.

**Table 15:** Condition setting of scenario deduction for the Nanjing COVID-19 epidemic.

Step	Conditions
1	Occurring events, SE2, SE3, SE4, SE6, SE7; and non-occurring events, SE1, SE5
2	Occurring event, PE7; and non-occurring events, PE1, PE2
3	Occurring events, PE3, PE4; and non-occurring event, PE6
4	Occurring event, PE6
5	Occurring event, PE2
6	Occurring event, PE5; and non-occurring event, PE7

**Table 16:** Prediction probabilities for each scenario.

	Step 0	Step 1	Step 2	Step 3	Step 4	Step 5	Step 6
SE1	0.53	0	0	0	0	0	0
SE2	0.60	1	1	1	1	1	1
SE3	0.80	1	1	1	1	1	1
SE4	0.65	1	1	1	1	1	1
SE5	0.50	0	0	0	0	0	0
SE6	0.55	1	1	1	1	1	1
SE7	0.45	1	1	1	1	1	1
PE1	0.48	0.0576	0	0	0	0	0
PE2	0.70	0.7326	0	0	0	1	1
PE3	0.65	0.7627	0.2312	1	1	1	1
PE4	0.55	0.9003	0.7412	1	1	1	1
PE5	0.50	0.9779	0.9049	0.9195	0.9869	0.9992	1
PE6	0.55	0.3246	0.1613	0	1	1	1
PE7	0.35	0.0767	1	1	1	1	0
RE1	0.78	0.9998	1	1	1	1	1
RE2	0.25	0.0177	0.0528	0.0097	0.0033	0.0044	0
RE3	0.35	0.4730	0.8376	0.8286	0.6773	0.0772	0.0800

## Data Availability

The datasets generated and analyzed for this study can be requested from the correspondence authors at ewang01@esf.edu (Wang, E.) and 6370@njtech.edu.cn (Li, L.).
